# Adaptation of the Stakeholders’ Walkability/Wheelability Audit in Neighborhoods (SWAN) Tool for Individuals With Diverse Disabilities: Protocol for a Mixed Methods Study

**DOI:** 10.2196/60553

**Published:** 2025-04-10

**Authors:** Atiya Mahmood, Farinaz Rikhtehgaran, Rojan Nasiri, Niloofar Hedayati, Sepehr Pandsheno, Aislynn Sharrock, Diana Juanita Mora, Sogol Haji Hosseini, François Routhier, W.Ben Mortenson

**Affiliations:** 1 Department of Gerontology Simon Fraser University Vancouver, BC Canada; 2 Urban Studies Program Simon Fraser University Vancouver, BC Canada; 3 Faculty of Health Sciences Simon Fraser University Burnaby, BC Canada; 4 School of Rehabilitation Sciences Faculty of Medicine Université Laval Quebec, QC Canada; 5 Centre for interdisciplinary research in rehabilitation and social integration, Centre intégré universitaire de santé et de services sociaux de la Capitale-Nationale Quebec, QC Canada; 6 Department of Occupational Science & Occupational Therapy Faculty of Medicine University of British Columbia Vancouver, BC Canada

**Keywords:** age and accessibility, disability experiences, community engaged research, inclusive urban design, user-led built environment audits

## Abstract

**Background:**

The prevalence of sensory, cognitive, and mobility disabilities in Canada underscores the need to address environmental barriers. This study adapts and validates the Stakeholders’ Walkability/Wheelability Audit in Neighborhoods (SWAN) tool to assess the challenges the built environment poses for individuals with disabilities, aiming to inform policy changes for accessibility and inclusivity.

**Objective:**

This study aims to (1) adapt the SWAN tool for those with hearing, vision, or cognitive disabilities; (2) validate SWAN tool for researching environmental barriers for people with disabilities, including older adults; and (3) offer insights for policy changes in the built environment, contributing to literature and guiding future research.

**Methods:**

The study uses a community-based research approach, carried out over 4 phases within an 18-month period in British Columbia. Phase 1 includes adapting and pilot-testing of the SWAN tool. In Phase 2, street intersections are identified for data collection using Geographic Information System tools and consultations with municipal officials. Phase 3 involves recruiting participants across four disability categories. The final phase includes analyzing the data and disseminating findings.

**Results:**

Data collection concluded in September 2024, involving 80 eligible participants across four streams in preidentified hotspots. The results are expected to be published in March 2025. To date, data collection is ongoing, and we are currently in the process of data analysis.

**Conclusions:**

This study will contribute to the growing body of research on built environment accessibility by adapting the SWAN tool for individuals with diverse disabilities. By identifying key barriers in urban spaces, the study aims to inform policy changes that will lead to more inclusive, accessible, and safe urban environments for all individuals.

## Introduction

### Overview

The number of people living with sensory (hearing and vision), cognitive, and mobility disabilities is increasing in Canada. In 2019, approximately 5% of people aged 15 years and older had a hearing disability, and in 2020, a total of 567,000 people were living with a cognitive disability [1]. About a quarter (24.1%) of the population living with disabilities are aged 65 years and older [2].

Mobility restrictions are not typically the result of a single cause but arise from an interaction of risk factors in various domains, both individual and environmental [3]. Historically, disability research primarily relied on the medical model, emphasizing the individual and their specific impairments or conditions [4]. However, in more recent times, disability models have shifted their focus toward understanding the dynamic interplay between individuals and their surrounding environment [5]. Environmental characteristics are hypothesized to limit or promote an individual’s ability to complete purposeful actions and fulfill role expectations, affecting physical functioning and disability [3]. Lawton [6] proposed several dimensions of environment that are important for older adults: personal environment (family and friends), suprapersonal environment (ie, neighborhood racial or age composition), social environment (norms or values related to society or culture), and physical environment (eg, built environment). The physical environment is defined as the human-made or human-altered space in which individuals live out their daily lives [7] and is the focus of this paper. The built environment has a profound impact on the mobility of older adults and people living with disabilities, which can affect their health and quality of life [3,8-11]. This aligns with the International Classification of Functioning, Disability, and Health, which posits disability and functioning as outcomes that result from the interplay between health conditions (such as diseases, disorders, and injuries) and contextual factors, including the built environment.

For instance, individuals who are deaf or hard of hearing (DHH) feel less safe when navigating the pedestrian environment as they struggle to hear traffic on the road [12,13]. Persons living with mild cognitive impairment (MCI) often experience challenges with navigation of their own environment, which is heightened when communities do not integrate sufficient green spaces and landmarks (such as large shops, libraries, community centers, and senior centers) to help reduce the stress of wayfinding [14]. Moreover, people using mobility assistive technologies (MAT) may find it challenging to navigate the built environment, especially during rain and snow because of inadequate drainage or snow removal [10]. People with vision impairment (VI) also face difficulties due to the lack of accessibility in public spaces and transportation systems [11].

Limited research exists on the role of environmental factors on mobility and social participation of individuals with disabilities, creating a gap in the literature [3,8-11]. Environmental variables that can affect the experience of being mobile in a place, fall into 2 broad categories: macroscale, consisting of structural features such as street interconnectivity and land use mix [15,16]; and microscale, or details, such as aesthetics and sidewalk design, and maintenance [17]. While much research has concentrated on macroscale variables that define walkability [18], investigating microscale features is also valuable for understanding the mobility experience [19-22]. Microscale characteristics of the built environment can often be modified at a lower cost and within a shorter timeframe compared with restructuring macroscale designs [23].

Various audit tools have been developed and tested to evaluate the microscale qualities of the built environment, particularly at the street level, through on-site visits [24]. The Stakeholders’ Walkability/Wheelability Audit in Neighborhoods (SWAN) is a microscale, user-led audit tool designed to evaluate both objective and subjective aspects of the built environment that affect the lives of older adults and individuals using mobility assistive devices, persons who are DHH, and persons living with MCI including dementia [25,26].

The SWAN tool is an adaptation of the SWEAT-R tool that captures the perspective of persons with disabilities [25]. Moreover, the development of the SWAN tool included a comprehensive literature review and incorporated aspects of other user-led tools, such as the Microscale Audit of Pedestrian Streetscapes [23,27], the Built Environment and Active Transport Neighborhood Assessment [28], and Jane’s Walk: Walkability Checklist [29].

Previous research using the SWAN tool was primarily done with individuals with mobility disabilities using MAT [30]. To incorporate a wider variety of disability experiences, the SWAN tool has been adapted to accommodate individuals’ living sensory disability (hearing and vision) as well as those with cognitive disabilities, including early stages of dementia and MCI. This adaptation enables these populations to systematically evaluate their neighborhoods.

Using a community-based participatory research approach, the SWAN tool was developed in collaboration with a committee of individuals with lived and professional experience, ensuring that diverse perspectives and needs are integrated into the research process. This approach promotes self-advocacy among participants and facilitates policy changes that are reflective of community needs [31].

### Objectives

This study aims to (1) adapt the mobility tool for individuals living with hearing or vision as well as cognitive disability; (2) validate the tool for researching barriers in the built and social environment for persons with disabilities, including those with vision, hearing, cognitive, and mobility disabilities (including older adults); and (3) provide insights for decision-making and policy changes in modifying the built environment.

This protocol paper aims to contribute to existing literature and guide future studies on how individuals with cognitive, sensory, or mobility disabilities navigate the built environment. The goal is to fill knowledge gaps and provide evidence-based results for municipalities and communities to implement necessary policy changes for a safe and accessible living environment.

## Methods

### Overview

The research will be conducted over 4 phases within an 18-month period in British Columbia. Advisory committees including individuals with a variety of disabilities have been created to ensure the consideration of inputs or concerns of these individuals in the research project through a participatory research approach. These committees include individuals with mobility, visual, and hearing disabilities and early-stage dementia. The committees meet 2 to 3 times a year to provide feedback on the ongoing phases of the research project, which are presented below.

The first phase involves conducting a literature review, tool consolidation, and pilot-testing. Phase 2 entails identifying street intersections for data collection using Geographic Information System layers and discussions with municipal officials. Phase 3 involves collecting data across various streams of disabilities. The fourth phase includes data analysis and knowledge mobilization efforts.

### Ethical Considerations

This study has been reviewed and approved by the ethics boards of Simon Fraser University and the University of British Columbia (H21-01234). To protect identities, names and any other information that might identify a participant will be removed from transcriptions and field notes. Any photos taken as part of data collection that could potentially identify individuals will be blurred. All data will be collected, managed, and stored in accordance with university research ethics procedures, and all data will be anonymized. We perceive that the risks for physical or emotional harm to the participants associated with the proposed research are minimal. The time and effort required by participants is minimal, and there is no deception or other manipulation of participants. Given that participants might share difficult experiences, which may lead to emotional and/or psychological distress, the researcher will make clear at the beginning and throughout the interview that participation is voluntary, and participants can withdraw their consent at any time without harm. Participation in this study is voluntary, and participants can decide to opt out. An honorarium of CAD $75 (approximately US $53) will be provided to participants.

### Phase 1: SWAN Tool Development Process

#### Overview

To conduct the walking/wheeling audits with DHH individuals and those living with cognitive and vision disabilities, the original SWAN tool (that is, SWAN for MAT users) was adapted through reviewing the literature and consultations with persons with disabilities or persons with professional experience. Involving persons with disabilities and professionals with relevant experience fosters a more inclusive approach, ensuring that the tool reflects the diverse perspectives within the community. The incorporation of findings from both academic and gray literature allows for evidence-based adaptations, increasing the tool’s validity and reliability for the target populations.

#### Literature Review

Relevant concepts pertaining to DHH individuals and those living with cognitive and vision disabilities, focusing on their experiences and interactions with the safety and accessibility of the outdoor built environment, were taken from reviews done by our study team [10,25,32,33]. This allowed for collating similar concepts to pinpoint areas where the tool may need expansion or clarification to better support these specific populations.

#### Content Comparison and Tool Consolidation

After developing the newly adapted tools, they were charted in Microsoft Excel alongside the original SWAN tool for content comparison, sequencing of questions, and language simplicity and consistency. Upon review of all tools, they were finalized as the Hearing and Mobility Tool, the Dementia Tool, and the Vision Tool.

#### Pilots

To test the functionality of the tools in the field, 4 pilots were conducted with persons with disabilities from the 4 populations of interest. These pilots served as an opportunity to try the tools with participants and gather feedback on the questions, content, process, and flow of data collection. Since data collection involves a walking/wheeling method, both the research team and participants experience this process in the field, allowing for adjustments to be made accordingly. During the pilot sessions, particular attention was given to ensuring the clarity and comprehensibility of the terminology used in the tools. This was done to ensure individuals with different levels of abilities could easily understand the features being inquired about.

Following the pilot session with individuals with vision disabilities, substantial adjustments were implemented to improve the tool’s readability. These changes encompassed modifications to align the tool with the Canadian National Institute for the Blind standards, ensuring adherence to best practices for accessibility in both design and functionality. These adjustments, informed by participant feedback and collaboration with the Canadian National Institute for the Blind standards, are aimed at fostering a more user-friendly and inclusive experience for individuals with vision disabilities during data collection.

### Phase 2: Identification of Data Collection Locations Through Community and Research Project Partnerships

#### Overview

To identify areas for data collection, the research team undertook a stepwise approach, namely (1) generating prioritized sites and (2) hosting an interactive community forum to finalize locations.

#### Intersections for Data Collection

The research team identified data collection areas in 6 partner municipalities across Metro Vancouver using open-source data and ArcGIS. They focused on pedestrian-involved collisions data, integrating additional layers such as transportation hubs (eg, sky train stations) and city center locations to create maps and identify “hotspots” for data collection. The outcome was maps highlighting 7 to 10 intersections in each municipality. Subsequently, the team met with municipal officials to discuss and prioritize 3 to 4 intersections for the data collection per municipality based on their feedback and guidance.

#### SWAN Community Forum

The second step involved hosting an interactive forum with persons with disabilities, community partners from senior centers, and municipal officials. The goal was to understand challenges at intersections and surrounding areas, examining barriers and facilitators to mobility. The forum also explored designing interventions based on evaluating these areas in relation to municipal priorities and funding.

### Phase 3: Participant Recruitment, Coordination, and Data Collection

#### Overview

Finalizing the tool and confirming selected intersections enables the research team to proceed with participant recruitment for the 4 populations: individuals with cognitive, mobility, hearing, and vision disabilities, starting with those living with cognitive disabilities. In this study, we aim to collect data from 80 participants across various disability categories. The minimum sample sizes for each category are as follows: 30 participants using MAT, 15 individuals with MCI, 15 individuals who are DHH, and 20 individuals with VI. These numbers are selected to ensure sufficient data for validity and reliability tests. The larger sample size for participants using MAT reflects their greater prevalence compared with the other groups, allowing for a more comprehensive analysis of the effects of MAT in our study. All data collection will be concluded by September 2024. While our recruitment methods are committed to diversity and inclusion, we recognize the importance of providing specific details on participant demographics. By including a diverse range of participants in terms of age, gender, ethnicity, and disability type, we aim to gain a comprehensive understanding of the barriers and facilitators present within the built environment.

#### Recruitment

The research team will recruit participants from community centers, relevant organizations serving persons with disabilities, and health care connections. This ongoing process targets DHH individuals, those with hearing and vision disabilities, those with early stages of dementia or MCI, and MAT users. To streamline participant onboarding, a researcher will act as the central administrator. This individual will verify eligibility, review study details, consent, web-based online tool training, and COVID-19 protocols with participants before scheduling data collection sessions. Sessions will be scheduled based on participant and research team availability. The lead research assistant (RA) for each session will maintain communication with participants and ensure all necessary documents (SWAN tools, consent forms, and equipment) are ready for the efficient completion of the audits.

#### On-Site Preparation Research Team

On the day of data collection, the lead and accompanying RAs will arrive in advance to assess the presence and accessibility of public washrooms. Additionally, they will identify either an indoor or covered location for the completion of the SWAN Secondary Observation Form (SOF). This form will be filled out in the format of a 15- to 20-minute qualitative interview.

#### On-Site Preparation Participant

Before starting the audit with the participant, the lead RA will review the consent form to ensure the participant understands the study objectives, the use of collected data, and their right to withdraw. Next, a demographic form will be completed to contextualize the participant’s answers. The participant will be oriented on the path of travel using a map of the intersections to be covered. The lead RA will accompany the participant throughout the walking/wheeling audit, providing assistance as needed or taking a more active role by reading questions aloud, based on the participant’s preference.

#### SOF Content

The SOF includes open-ended questions similar to those in the SWAN tool, encouraging discussion and reflection from the participant. Responses will be either audio recorded or handwritten based on the participant’s consent choice. By incorporating the secondary observation interview guide, the SWAN tool gains a more comprehensive understanding of participants’ interactions with their neighborhoods.

#### Home and Community Environment Survey

During the data collection phase, 2 complementary tools will be used: the Home and Community Environment (HACE) tool and the SWAN tool. Participants can either complete these tools themselves or have assistance from a secondary RA. This dual approach enhances the validation process, providing a more thorough assessment of the research variables.

The HACE tool, designed as a self-report measure, will be directly administered by the participants. It aims to evaluate various factors within an individual’s HACE that might influence their level of community participation. In this study, the HACE tool serves as an additional validation measure for the effectiveness of the SWAN tool [34].

The initial HACE prototype consisted of 44 items assessing the physical, attitudinal, and political aspects of HACEs. Specifically, questions related to the community mobility domain will be included in the SWAN project’s data collection process.

#### Sidewalk Accessibility Index

The Sidewalk Accessibility Index (SI) serves as an indicator to assess the performance of sidewalks and public spaces, focusing on the needs and expectations of wheelchair users to define accessible routes within urban road networks. It considers various variables that contribute to the comfort and safety of wheelchair users, weighted according to their perceptions [35].

In the SWAN project, the SI will be used as a complementary tool for validation purposes. During data collection, a secondary RA completes the SI, which gathers data on 4 key aspects related to sidewalks: evenness, maintenance, width, and surface quality. However, the SWAN project focuses solely on these sidewalk-specific factors and does not inquire about the suitability of pedestrian crossings.

Sidewalks and public spaces should provide an environment that meets the needs of all users, ensuring comfort and safety regardless of physical limitations, whether temporary or permanent. The SI variables are designed to describe aspects of comfort and safety related to pedestrian movement along the block and crossing street intersections.

#### Finalization of Data Collection

The participant will then be provided with an honorarium for their time spent, and if required, a discussion of a second data collection will take place to complete any outstanding segments. The lead RA is responsible for ensuring the completion of all documents along with uploading and storing data collected appropriately. Finally, a reflection form will be completed by the researchers to reflect on the data collection.

### Phase 4: Data Analysis

#### Overview

The analysis will focus on validating the SWAN tool, which collects both quantitative and qualitative data. Objective and subjective scores will be calculated for its 5 domains. In order to verify the validity of the SWAN tool, 2 additional audit tools will be used to collect data, including HACE [34] and SI [35]. Walk score results for the audited area will also be compared with the SWAN result for further validation. Details on tool validation methods can be found in the section on tool validity. Additionally, the interrater reliability (IRR) of the SWAN tool will be assessed. More information is provided in the IRR section.

Quantitative data from SWAN and other tools will be entered and organized in Microsoft Excel. This includes coding and scoring based on the codebook and calculating domain scores within Excel. For further analysis, the R programming language in RStudio (Posit, PBC) will be used for IRR and tool validity assessments. Qualitative responses from the SOF will be entered and analyzed in NVivo software (Lumivero). More details on SOF analysis can be found in the *Qualitative Data Analysis* section. The specific steps of data analysis are outlined in the following sections.

#### Data Cleaning and Reorganization

After data are entered and prior to moving forward with the analysis, certain questions will be moved to their original domains. The questions were moved to a different domain for efficient data collection. Although they were physically sequential on site, they are better organized in a separate domain for analysis. For example, questions about street safety features were placed in the function of the street crossing domain during data collection but belong to the safety domain.

#### Weighting of Scores

The primary step in calculating scores for street segments involves assigning weights to both the domains and subdomains, ensuring a total cumulative score of 100 for all domains. These weights are determined through a comprehensive literature review and expert recommendations.

For individuals who are DHH, use medical assistance technology, and have cognitive disabilities, the domain weights remain consistent. However, there is a slight variation in the vision stream weighting, reflecting the unique needs of individuals with visual impairments. Specifically, the “appearance and maintenance” domain is considered less critical. Therefore, the vision stream receives a weight 5 points lower than other streams, which is then added to the “sidewalk functionality” subdomain.

“Functionality” and “safety” are deemed the most crucial domains for neighborhood accessibility for individuals with disabilities. Consequently, each of these domains will be assigned the highest weight. As shown in Table 1, functionality is divided into crossing functionality (20 points) and sidewalk functionality (15 points in vision stream, 10 points in other streams). The same weights will apply to safety subdomains, traffic safety at 20 (covering pedestrian and vehicle interaction safety), and personal safety at 10 (focusing on subjective safety perceptions).

**Table 1:** Domain and subdomain weights.

Domains and subdomains		Vision stream	Other streams
**Functionality**		30	35
	Crossing functionality	20	20
	Sidewalk functionality	10	15
**Safety**		30	30
	Traffic safety	20	20
	Personal safety	10	10
Land use and supportive features		20	20
Appearance and maintenance		15	10
Social aspect		5	5
Total		100	100

The “land use and supportive features” domain, recognized as the second most critical, will receive a weight of 20. “Appearance and maintenance” will be weighted at 10 for vision and 15 for all other streams, due to overlapping questions within the land use and supportive features domain. In contrast, the “social aspects” domain, with a limited set of 5 questions focusing on subjective assessments, will have the lowest weight.

Subsequently, a detailed review of questions within each domain and subdomain will extract essential concepts and elements. The assigned weight will then be evenly distributed among these identified concepts and elements to avoid undue emphasis on specific concepts. For instance, the 20 points allocated to the subdomain of crossing functionality will be evenly divided among concepts (eg, curb ramp, crosswalk, and pedestrian signal) within that subdomain. Finally, the weight assigned to each category of questions will be equally distributed among all questions within that category to ensure a balanced weighting process.

#### Objective Scores

There will be 3 steps to calculate the objective score for each domain. First, responses to questions will be converted into numeric codes based on the code book. Second, these codes will be multiplied by the question’s weight to calculate the question’s score. Third, the scores for all questions within a domain will be summed and divided by the maximum possible score for that domain. To make the aggregated objective score easier to understand, it will be multiplied by 100% for each domain.

Some questions in the SWAN tool are reverse-coded to avoid confusion. For instance, a positive response to the presence of a certain physical feature indicating a barrier (eg, “transition from the curb ramps into the crosswalk causes problems”) would be scored as “1,” though it is not a facilitator in this context. These questions were reverse-scored as needed to ensure the total score includes only true “Yes” scores.

In addition to the final objective score for each domain, a total score for the SWAN tool will be calculated by averaging the scores for each domain. This total score will facilitate comparisons between different audited segments.

#### Codebook

In the SWAN tool, response options typically include “Yes,” “No,” “Don’t Know,” and “Not Applicable,” as shown in Table 2. However, for individuals with vision disabilities, an additional option, “Cannot detect,” is provided for cases where the participant cannot clearly see the object but is aware of its existence. When participants with vision disabilities choose this option, it is treated as equivalent to selecting “No,” as the object is not detectable or functioning properly for the user. During the coding process, a value of “1” will be assigned for the presence of an assessed environmental feature that enhances walkability/wheelability, and “0” for its absence. Reverse-coded questions, such as “The outdoor patio(s) is/are an obstacle to walking/wheeling,” will use “0” to represent the presence of this barrier and “1” to denote its absence.

**Table 2:** Different types of questions and scoring rationale (missing data: n=99).

Type of question and possible responses		Code
**General questions**		
	Yes	1 (0 in case of reverse coding)
	No (cannot detect, specifically in the tool designed for vision impairment)	0 (1 in case of reverse coding)
	Not applicable	98
	Don’t know	97
**Both sides questions**		
	Both sides	2 (0 in case of reverse coding)
	One side	1
	Neither side	0 (2 in case of reverse coding)
	Not applicable	98
	Don’t know	97
**Directional questions**		
	One-way	1
	Two-way	0
	Not applicable	0
	Don’t know	0

For items referring to “one side or both sides” of a street segment, they will be grouped together as a scale. For example, “Are there curb ramps/cuts present?” will be coded as “2” if they are present on both sides, “1” if present on only one side, and “0” if not present at all. The same approach applies to items with a positive or negative impact on walkability/wheelability.

In addition to general and 2-sided questions, the SWAN tool includes questions about the direction of traffic, with responses like “one-way” and “two-way”. Through a disability lens, “one-way” responses will be coded as “1” and “two-way” as “0” since crossing one-way streets is less complex.

Responses like “not applicable” and “don't know,” based on the data collection area and participants’ familiarity, will be excluded from the scoring system to ensure it accurately reflects walkability/wheelability factors.

#### Subjective Ratings

Participants in the SWAN tool provide subjective ratings using a 5-point Likert scale at the end of each domain. This scale ranges from “poor” (scored as “1”) to “excellent” (scored as “5”), reflecting their subjective experience of the assessed domain. These ratings are averaged and converted into percentages to indicate participants’ subjective perceptions of the audited segment. Comparing these subjective scores with the objective scores may reveal interesting findings.

#### Qualitative Data Analysis

As indicated, the qualitative data will be collected using the SOF which contains open-ended questions based on the 5 primary domains, and 4 subdomains in the SWAN tool. Qualitative data gathered using the SOF is intended to capture detailed insights from the participants regarding the built environment and their lived experiences. For instance, within the personal safety subdomain, participants will be asked the following “Are there things that make you feel safe or unsafe when walking/wheeling during the daytime?” Collectively, the SOF data and the photo commentary build the qualitative dataset connected to the SWAN tool.

The qualitative data will be analyzed by using both Braun and Clark’s [36] seminal multistep thematic analysis process and by drawing on later advances in their methodological framework [37]. Their original analytical process consists of six integrated phases including (1) data familiarization and review, (2) code development and comparison, (3) code consolidation and early theme exploration, (4) theme confirmation and mapping, (5) theme refinement and name creation, and (6) research findings write-up (Braun and Clark [36]). While the open-ended questions contained in the SOF are grounded in the conceptual domains of the SWAN tool, the qualitative analysis process will be both inductive and reflexive [36,37]. Therefore, the analysis will be guided by the data itself [36] and will aim to identify insights and interpretations of the data beyond known built environment barriers and facilitators while also embracing the subjectivity of the research team members [37]. The research team will also independently and collectively engage in reflexive journaling practices and group discussions throughout the qualitative analysis process [38]. These practices will help the research team examine their subjectivity and understand its connection to their analysis [39]. Last, this will also contribute to the quality of the analysis; specifically, the adoption of these reflexive methods will contribute to the equitable and fair representation of all participant perspectives [38].

#### Step 1: Data Familiarization and Review

The raw qualitative data will undergo initial transcription and cleaning using Otter.ai. Subsequently, the transcriptions will also be verified by several RAs. This process will not only ensure a clear and reliable dataset for analysis but will also help the research team familiarize themselves with the data.

#### Step 2: Code Development and Comparison

After the data have been transcribed and cleaned the coding process will begin. This will include the development of early/open codes and exploration of the codes across transcripts [36]. Coding will be undertaken by individual research team members, however, will be complemented by group discussions to allow for exploration of the research team’s subjectivity and interpretation of the data.

#### Step 3: Code Consolidation and Early Theme Exploration

Following the coding of the transcripts, the research team will work to consolidate the existing codes and explore relevant themes identified within the data [36]. This process will be collaborative as all research team members will aid in collating existing codes, and in the development of a preliminary thematic map/diagram.

#### Step 4: Theme Confirmation and Mapping

After the potential themes have been identified, the research team will work to solidify the themes. This will involve examining if the preliminary themes fully represent and reflect the data [36]. This process will be iterative and will potentially involve revisiting the coding process. This stage will not conclude until all team members agree that the analysis fully captures the data, and a cohesive thematic map has been developed.

#### Step 5: Theme Refinement and Name Creation

The research team will then work to ensure each theme is well-defined and succinct [36]. This process will also be iterative and will work to identify the relevance of each theme in relation to the broader goals of the SWAN tool.

#### Step 6: Write-Up of Research Findings

In the final step, the team will elaborate on each theme, discussing their significance with evidence from the data, including quotes. This comprehensive write-up will aim to convey theme frequency, and importance, within the research context.

#### IRR Analysis

The reliability of the SWAN will be determined by calculating IRR using the paired observer method. The IRR will compare the objective scores of older adults or persons with disability and the secondary RA. This will be calculated both in percentage agreement and Cohen κ to compare the results [40]. Percentage agreement is a straightforward measure that calculates the proportion of agreement between 2 observers as a percentage of the total observations; however, in 1960, Cohen [41] critiqued the use of percent agreement due to its inability to account for chance agreement. He introduced the Cohen κ, developed to account for the possibility that raters actually guess on at least some variables due to uncertainty [40]. Cohen suggested the κ result be interpreted as follows: values ≤0 as indicating no agreement, 0.01-0.20 as none to slight, 0.21-0.40 as fair, 0.41-0.60 as moderate, 0.61-0.80 as substantial, and 0.81-1.00 as almost perfect agreement [41].

#### Tool Validity

To validate the SWAN tool, correlation analyses will be conducted between its domains and other environmental audit tools used in data collection: HACE, SI, and Walkscore. HACE and SI scores will be normalized using formulas outlined in their respective papers [34,35], while Walkscores for the audited area will be obtained via web. After examining data distribution, the appropriate correlation analysis method, Pearson correlation, will be applied to assess associations between scores from different tools. Pearson correlation lies between –1 and 1. Values near 0 mean no (linear) correlation and values near SD 1 mean very strong correlation. The negative sign means that the 2 variables are inversely related, that is, as one variable increases the other variable decreases. The following table gives a guideline on the strength of the linear relationship corresponding to the correlation coefficient value [42].

As no comprehensive audit tool similar to SWAN was identified in existing literature, each additional audit tool was purposefully selected to assess the validity of specific SWAN domains. Therefore, SI will be compared with functionality, HACE with safety, and Walkscore with land use and supportive features. The matrix in Table 3 illustrates which SWAN domains or subdomains will be compared with measures from HACE, SI, and Walkscore tools.

**Table 3:** Similarity between the Stakeholders’ Walkability/Wheelability Audit in Neighborhoods (SWAN) domains and subdomains and other tools.

Extra tools and measures used for tool validation		Domains and subdomains of the SWAN tool										
		Functionality			Safety			Land use and supportive features		Appearance and maintenance		Social aspect
		Street crossing	Sidewalk	Traffic safety		Pedestrian safety						
**SI^a^ (sidewalks)**												
	Even		✓			✓						
	Well-maintained		✓			✓			✓			
	Surface condition		✓			✓			✓			
	Width		✓									
	Intersection	✓		✓		✓						
**HACE^b^**												
	Uneven sidewalks		✓			✓						
	Easy to use sidewalks		✓			✓						
	Safe	✓	✓	✓		✓						
	Places to rest										✓	
	Curb ramps	✓		✓								
**Walkscore**												
	Access to amenities						✓					

^a^SI: Sidewalk Accessibility Index.

^b^HACE: Home and Community Environment.

## Results

Data collection concluded in September 2024, involving 80 eligible participants across 4 streams in preidentified hotspots. The results are expected to be published in March 2025. To date, data collection is ongoing, and we are currently in the process of data analysis.

## Discussion

### Expected Findings

By focusing on a diverse group of participants, including those using MAT, those living with MCI and VI, and those who are DHH, the study aims to provide a comprehensive understanding of the barriers and facilitators present within the built environment using the SWAN tool. As there are no validated tools to capture this population perspective while navigating the built environment, the anticipated findings are expected to contribute to the existing literature by validating the SWAN tool in diverse disability categories. We hypothesize that similar to prior research, functionality and safety will emerge as key factors influencing accessibility across all disability groups. Past studies have consistently shown that features like sidewalk width, smoothness, and the presence of safe crossings are fundamental to pedestrian mobility [3,8-11]. However, our approach considers physical features, by integrating subjective participant ratings and qualitative data collected through the SOF to capture subjective experiences. This dataset should provide a more comprehensive view of how the built environment influences community participation and social participation for individuals with disabilities.

A key strength of this study is its comprehensive inclusion of multiple disability types. This research adopts a holistic perspective, capturing the full spectrum of disability-related accessibility challenges.

One important aspect that was not fully addressed in this study was the inclusion of individuals who are deaf-blind. This population, which experiences both vision and hearing impairments simultaneously, faces unique challenges that were not directly captured in this phase of the research. Due to the complexity of their needs, the lack of comprehensive accessibility tools for this group, and the potential for logistical difficulties in coordinating data collection with deaf-blind individuals, this population was excluded from this study. However, their experiences are equally critical to understanding how the built environment affects individuals with multiple disabilities.

To ensure that the findings from this study reach a wide audience and have a meaningful impact, we have developed a comprehensive knowledge mobilization strategy. A central element of this plan is the community forum, which will provide an interactive space for people with disabilities, urban planners, local government officials, and other key stakeholders to engage directly with the research findings. This forum will facilitate the exchange of experiences and insights from individuals with disabilities, offering a platform to share their lived experiences navigating urban spaces. Through this collaborative dialogue, we aim to raise awareness and generate actionable recommendations for improving the accessibility of urban environments.

Additionally, as part of the knowledge mobilization strategy, we will develop multimedia resources including games and videos to engage both the disability community and the general public in the study’s outcomes. The games will simulate the various mobility challenges faced by individuals with disabilities, offering an immersive experience for users to understand and empathize with the barriers these individuals encounter. These interactive tools will be used as educational resources in community centers, schools, and public awareness campaigns to foster greater understanding and empathy.

### Conclusion

This study introduces a new approach to assessing urban accessibility for individuals with various disabilities using the SWAN tool; by adopting a community-based methodology, the research emphasizes the importance of including diverse disability perspectives in evaluating the built environment. The anticipated findings will provide valuable insights into the barriers and facilitators that impact mobility, safety, and overall accessibility in urban spaces.

While the study’s limitations include the exclusion of individuals who are deaf-blind and the focus on specific urban areas, these gaps present opportunities for future research. Expanding the participant pool and environments will strengthen the generalizability of the findings.

This work contributes new knowledge to the field by offering a comprehensive framework for urban accessibility that integrates both subjective and objective measures. It underscores the importance of inclusive design and offers practical implications for urban planners and policymakers striving to create more accessible, inclusive communities.

## Abbreviations

DHH: deaf or hard of hearing
HACE: Home and Community Environment
IRR: interrater reliability
MAT: mobility assistive technologies
MCI: mild cognitive impairment
RA: research assistant
SI: Sidewalk Accessibility Index
SOF: Secondary Observation Form
SWAN: Stakeholders’ Walkability/Wheelability Audit in Neighborhoods
VI: vision impairment

## Data Availability

The datasets generated or analyzed during this study will be available from the corresponding author upon reasonable request.
